# Efficacy and safety of male fertility-sparing radical cystectomy with orthotopic neobladder versus radical cystectomy and nerve-sparing cystectomy: a meta-analysis

**DOI:** 10.3389/fonc.2025.1617812

**Published:** 2025-10-31

**Authors:** Yu Huang, Hongjin Shi, Qun Wang, Zhengyan Wang, Rui Wang, Yawei Zhang, Jiansong Wang, Nan Zhang, Shi Fu, Haifeng Wang

**Affiliations:** ^1^ Department of Urology, Kunming Municipal Hospital of Traditional Chinese Medicine, Kunming, China; ^2^ Department of Urology, The Second Affiliated Hospital of Kunming Medical University, Kunming, China; ^3^ Department of Urology, South Yunnan Central Hospital of Yunnan Province, Yunnan, China

**Keywords:** bladder cancer, radical cystectomy, modified, meta-analysis, orthotopic neobladder

## Abstract

**Background:**

Radical cystectomy (RC) serves as the gold standard treatment for organ-localized bladder cancer; however, postoperative complications diminish the quality of life of patients. Whether male fertility-sparing radical cystectomy(FSRC) with orthotopic neobladder (ONB) surpasses RC and nerve-sparing cystectomy (NSC) remains controversial. The objective of this study is to compare the efficacy and safety of the two surgical approaches.

**Methods:**

In accordance with the PRISMA (Preferred Reporting Items for Systematic Review and Meta-Analysis) statement, PubMed, Web of Science, Embase, CNKI databases, Medline, and Cochrane Library were searched until June 2024. Eligible studies were identified in line with the inclusion and exclusion criteria.

**Results:**

A total of 10 studies encompassing 1104 patients were incorporated in this study. The outcomes demonstrated that fertility-sparing radical cystectomy (FSRC) presented significant superiority in erectile function (EF) (OR: 12.67; 95% CI 3.27-49.03; P<0.001), daytime urinary continence (OR: 5.91; 95% CI, 1.83-19.13; P = 0.003), and nocturnal urinary continence (OR: 5.13; 95% CI, 1.98-13.34; P<0.001) over non-fertility-sparing radical cystectomy (nFSRC). Compared with nFSRC, the incidences of postoperative prostate cancer (RD:−0.10; 95% CI, -0.21-0.10; P = 0.086), tumor local recurrence (OR:0.51; 95% CI, 0.26-1.00; P = 0.052), tumor metastasis (RD:-0.02; 95% CI, -0.09-0.06; P = 0.665) and 2-year survival (OR:1.21; 95% CI, 0.63-2.30; P = 0.567) after surgery were comparable. In the subgroup analysis, some differences in outcome measures were identified based on sample size, study type, control group, and study area.

**Conclusion:**

Under rigorous preoperative screening, male FSRC with ONB demonstrates certain efficacy and safety in the treatment of bladder cancer, particularly among younger patients, warranting broader clinical consideration. More relevant clinical RCTs are required.

**Systematic review registration:**

https://www.crd.york.ac.uk/PROSPERO/view/CRD42024558576

## 1 Introduction

Bladder cancer (BC) is a prevalent malignant urothelial tumor. It ranks ninth in terms of the incidence of BC worldwide, seventh among men, and thirteenth in terms of mortality (1). BC is predominantly urothelial carcinoma, and the majority of patients are already in muscle-invasive bladder cancer (MIBC) at the time of diagnosis. RC combined with bilateral pelvic lymph node dissection (PLND) constitutes the primary treatment for recurrent high-risk non-muscle-invasive bladder cancer (nMIBC) and MIBC (2), offering a sustained chance of cure, with the 5-year recurrence-free survival rate being 70% (3).

However, in standard RC, the related neurovascular bundles surrounding the prostate will be removed or damaged during the operation. This technique is associated with a considerable incidence and high prevalence of postoperative erectile dysfunction (ED), which may exert a significant influence on the quality of life, especially for younger patients (4, 5). In an attempt to enhance the quality of life of RC patients, Spitz devised and executed fertility-preserving cystectomy in 4 cases in 1989. By attaining a profound comprehension of the anatomical composition of the ejaculatory organ, the traditional surgical approach was modified, and all patients maintained erectile function (EF) after the surgery, among whom 3 cases had anterograde ejaculation and 1 case achieved procreation (6). Subsequently, Prostate capsule-sparing cystectomy (PCSC) was initially depicted by Schilling and Friesen in 1990 (7), subsequently reducing urinary incontinence and preserving EF. Variations exist in the management of male reproductive organs across different surgical approaches, with the primary objective being the preservation of the sexual nerve integrity to enhance postoperative EF. Under physiological conditions, urinary continence is maintained through coordinated action of the internal and external urethral sphincters (8), both of which are innervated by the pelvic autonomic nerves, including the cavernous nerves. Intraoperative damage to these neural structures may result in postoperative urinary incontinence. Nevertheless, oncological safety remains a critical concern. Some clinicians have expressed reservations that preservation of the prostatic capsule might facilitate tumor spillage from the bladder neck or urethral margin, thereby increasing the risk of local recurrence and distant metastasis (9, 10). The risk of prostate adenocarcinoma during prostate tissue-sparing cystectomy has also elicited concerns (11, 12). In this context, FSRC is defined as a surgical procedure involving RC and orthotopic neobladder (ONB), while preserving key male reproductive structures (such as the prostate gland, prostatic capsule, seminal vesicles, and vas deferens). Currently, FSRC can be approximately classified into the following categories (1): cystectomy with the preservation of the prostate capsule (2), cystectomy with the preservation of the prostate (3), cystectomy with the preservation of only the vas deferens and seminal vesicles (2, 9, 12–15).

Currently, some clinical investigations have attested to the favorable outcome of FSRC, and the effect of tumor control is comparable to that of RC (9, 16). Nevertheless, due to the variances in surgical procedures, disputes exist regarding efficacy and safety, and there is a dearth of unified standards that can be universally applied. We have also observed that some researchers have carried out systematic reviews and meta-analyses on the effectiveness of certain surgical methods. However, considering the limited number of controlled studies received and the absence of systematic and comprehensive analysis, it is infeasible to account for the differences in efficacy and safety between FSRC and nFSRC.

Therefore, a systematic review and meta-analysis of the efficacy and safety of FSRC with ONB versus nFSRC for BC was conducted to provide a better reference for clinical practice, to serve clinical treatment more accurately and effectively.

## 2 Methods

This review was prospectively registered in the PROSPERO database and reported following the PRISMA (Preferred Reporting Items for Systematic Reviews and Meta-Analyses) statement and AMSTAR (Assessing the methodological quality of systematic reviews) Guidelines (17, 18).

### 2.1 Search strategy

A literature search was conducted in PubMed, Web of Science, Embase, CNKI databases, Medline and Cochrane Library with the following search terms: ((Urinary Bladder Neoplasms) OR (bladder tumor) OR (bladder cancer) OR (bladder papilloma)) AND ((prostate sparing) OR (capsule sparing) OR (seminal vesicle) OR (erectile) OR (sexual OR ejaculation OR incontinence)) AND ((cystectomy) OR (cystoprostatectomy) OR (neobladder)) AND ((Randomized Controlled Trials) OR (Controlled Clinical Trial) OR (Observational Study)). Appropriate database-specific subject headings were employed where necessary. Results encompass articles published within the period from January 1, 2000, to June 1, 2024. Simultaneously, a manual search was also conducted from the references of relevant studies to broaden the search scope. Each included study was independently evaluated by two reviewers (Y.H. and H.S.), and any dissents were settled through consensus.

### 2.2 Inclusion/exclusion criteria

Studies meeting the following inclusion criteria were admitted (1): The study conducted a comparison between FSRC and RC or NSC (2). The types of clinical studies encompass randomized controlled studies, prospective controlled studies, and retrospective studies (3). The full text should contain at least one outcome parameter, such as postoperative sexual function, urinary control, tumor control, and survival (4). Language restrictions are limited to English and Chinese. The following studies were excluded: reviews, letters, case reports, low-quality research, and research with no detailed data.

### 2.3 Types of intervention and comparator

The intervention group comprised the following procedures (1): preservation of the entire or partial prostate tissue (2); preservation of the prostate capsule and adjacent periprostatic tissues (3); preservation of the seminal vesicles and vas deferens. In all three procedures, the relevant neurovascular bundles were preserved and collectively designated as FSRC, in accordance with the prior definition. The control group underwent either conventional RC or NSC, collectively referred to as nFSRC, as detailed in **Table 1**.

**Table 1:** Types of intervention and comparator.

		Part or the whole prostate	The capsule or peripheral part of the prostate	Seminal vesicles, vas deferens	Neurovascular bundles
Intervention group(FSRC)	PSC	✓	✓	✓	✓
	PCSC	×	✓	✓	✓
	SSC	×	×	✓	✓
Control group(nFSRC)	NSC	×	×	×	✓
	RC	×	×	×	×

PCSC, prostate capsule-sparing cystectomy; PSC, prostate-sparing cystectomy; SSC, seminal-sparing cysto-prostatectomy; NSC, nerve-sparing cystectomy; RC, radical cystectomy; ”√” denotes retention; ”×” denotes removal.

### 2.4 Data extraction

Our meta-analysis will extract the following data from each study (1): Basic information of the included studies: title, main author, sample size, publication year, and country (2); Research characteristics: research methods, subjects, interventions, and outcome indicators (3); Outcomes: postoperative urinary control, sexual function, oncology outcomes, etc. The data types in this study can be classified into binary variables and continuous variables. For instance, the outcome indicators depicting the urinary control and EF of patients as effective or ineffective, and the tumor recurrence and metastasis, the occurrence of prostate cancer (PCa), and the 2-year survival after surgery, which are counted based on the number of occurrences, are continuous variable data, and we converted them into binary variables. When continuous variables were reported in other forms in the main literature, we calculated the means and standard deviations (19).

### 2.5 Outcomes

The definitions of urinary incontinence and EF varied across the included studies and existing literature, with each outcome measure defined by the trial investigators to account for inconsistencies in data reporting. Main outcome measures (1): Definition of urinary control: Urinary incontinence was assessed by a valid questionnaire, the quantity of pads or self-impression report at 6 months after surgery, covering both daytime and nighttime (2). Sexual function was defined as sexual activity, EF, and ejaculatory function at 6 to 12 months after surgery, through validated questionnaires or self-impression reports based on preoperative and postoperative evaluations. The secondary outcomes were as follows: the oncologic outcomes were defined as any recurrence of local or metastatic diseases, the occurrence of PCa, and the number of overall survival beyond 2 years during the follow-up period.

### 2.6 Quality assessment

We assessed the quality of nonrandomized studies by employing the Newcastle-Ottawa Quality Assessment Scale (20). Studies were evaluated in three aspects: selection, comparability, and exposure/outcome. Studies were regarded as of high quality if they obtained at least 7 points. Given the presence of numerous confounding factors in retrospective studies, we excluded studies with a total score exceeding 7 points if the comparability between groups or the outcome assessment score was less than 2 points, to minimize potential bias and enhance the reliability of the study findings. The Cochrane Collaboration’s Risk of Bias Assessment Tool was utilized to evaluate randomized controlled trials (RCTs), and the risk of bias of each trial was classified into high, low, or unclear based on factors such as random sequence generation, allocation concealment, blinding of the study protocol by subjects and researchers, blinding of outcome assessment, incomplete outcome data, and selective reporting (21). All disputes were settled through discussions between the two commentators.

### 2.7 Statistical analysis

Statistical analysis was conducted using Stata 14.0 software. The weighted mean difference (WMD) and odds ratio (OR), along with 95% confidence interval (CI), were computed for continuous and dichotomous variables, respectively. When the outcome of interest was zero in either the experimental or control groups, the risk difference (RD) was adopted as the pooled statistic. The chi-square test and I^2^ test were employed to analyze the heterogeneity among studies. A random-effects model was utilized if there was significant heterogeneity (P > 50%); otherwise, a fixed-effects model was applied. Statistical significance was defined as P < 0.05. To further assess the robustness of the final results, sensitivity analyses were carried out.

### 2.8 Subgroup analysis

Based on the data extracted from the included literature, subgroups were defined according to region (Europe, Asia, and Africa), sample size (greater than 50 and less than or equal to 50), study type (RCT, retrospective, and prospective), and surgical control group (RC and NSC). Postoperative EF, daytime continence, nighttime continence, and postoperative PCa were analyzed.

### 2.9 Publication bias

Egger’s test and Begg’s test were employed to evaluate publication bias. In the event of publication bias, a trim and fill approach was utilized to estimate the missing studies and recalculate the results (22).

## 3 Results

### 3.1 Search results

**Figure 1:**
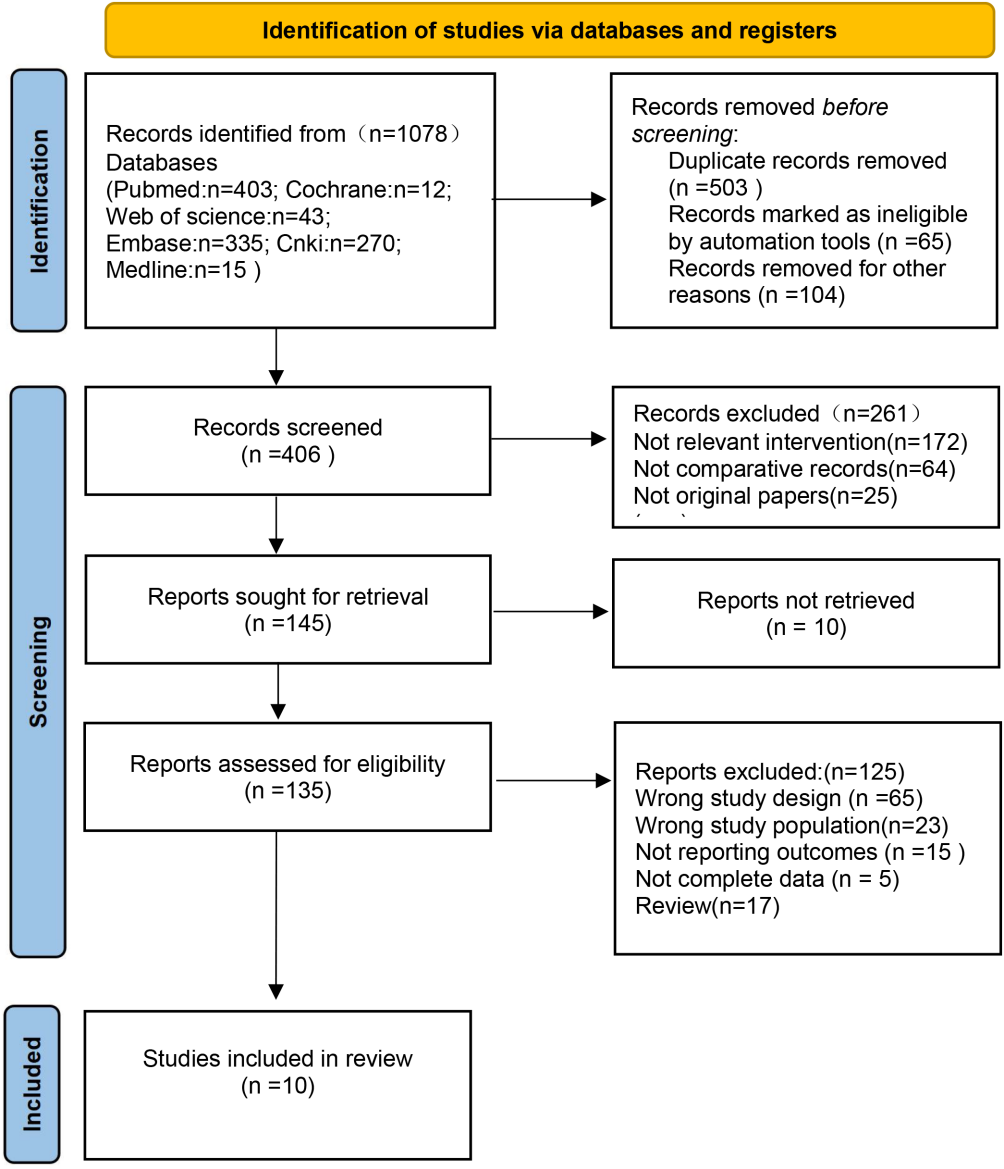
Flow diagram of studies identified, included, and excluded.

Our search strategy resulted in 1078 articles, among which 503 were duplicates. The rest of the articles were further screened in accordance with strict inclusion and exclusion criteria. Based on the title and abstract, 261 articles were excluded, and a total of 45 full-text articles were retained. Eventually, after eliminating irrelevant articles, a total of 10 articles were selected (**Figure 1**).

### 3.2 Characteristics of included studies

All studies included met the diagnostic criteria for BC and required RC. The reviewed studies comprised two RCTs and eight retrospective or prospective controlled studies. A total of 1, 104 patients were included in these studies, with 451 patients in the intervention group and 653 in the control group. Seven studies compared PCSC with RC or NSC, two studies compared PSC with RC, two studies compared SSC with RC or NSC, and one study compared both PCSC and SSC with NSC. The majority of patients were aged between 50 and 65 years, and the mean follow-up duration exceeded one year. Relevant data have been summarized in **Table 2**.

**Table 2:** Baseline characteristics of included studies and methodological assessment.

Study	Year	Region	Study type	Technique	Patients	Mean (standard deviation)		n(%)		NOS score
						Age	BMI	≥pT3	Neoadjuvant chemotherapy	
Abdelaziz et al.	2019	Africa	RCT	PCSC	45	62.2(5.8)	24.1(2.1)	NA	NR	–
				RC	51	63.7(6.8)	23.3(2.3)	NA	NR	
Jacobs et al.	2015	North america	RCT	PCSC	20	58(10)	30 (5)	2(10)	8(40)	–
				NSC	20	59(6)	31 (4)	4(20)	10(50)	
De Vries et al.	2009	Europe	Prospective	PSC	63	NA	NR	16(25)	NR	8
				RC	63	NA	NR	19(30)	NR	
Chen et al.	2017	Asia	Retrospective	PSC	14	57.5(13.93)	24.56(4.71)	NA	2(14)	7
				RC	11	61.55(15.03)	23.85(2.97)	NA	1(9)	
Basiri et al.	2012	Asia	Prospective	PCSC	23	59(14)	NR	NR	0(0)	8
				RC	27	61(12)	NR	NR	0(0)	
Colombo et al.	2015	Europe	Retrospective	PCSC, SSC	55	49.99(6.01)	NR	4(7)	0(0)	8
				NSC	35	58.1(5.34)	NR	3(8)	0(0)	
Wang et al.	2008	Asia	Prospective	PCSC	27	47(10.5)	NR	13(48)	NR	7
				RC	9	46(10.5)	NR	4(44)	NR	
Sadd et al.	2019	Europe	Retrospective	PCSC	60	NA	25(4)	18(60)	13(22)	8
				NSC	47	NA	26(3)	20(47)	11(23)	
He et al.	2022	Asia	Retrospective	PCSC	20	57.10(12.23)	23.09(2.93)	5(25)	1(5)	7
				RC, NSC	44	61.34(10.86)	23.91(2.85)	9(20)	6(14)	
Furrer et al.	2021	Europe	Retrospective	SSC	124	61.33(9.87)	26.67(4.07)	27(22)	15(12)	8
				RC	346	64.33(8.5)	27(4.37)	118(34)	60(17)	

PCSC, prostate capsule-sparing cystectomy; PSC, prostate-sparing cystectomy; SSC, seminal-sparing cysto-prostatectomy; NSC, nerve-sparing cystectomy; RC, radical cystectomy; NOS, Newcastle–Ottawa Quality Assessment Scale; RCT, randomized controlled trial; NA, not available; NR, not reported.

### 3.3 Risk of bias

Two RCTs were assessed for risk of bias using the Cochrane Collaboration’s tool, with the results presented in **Table 3**. Jacobs (23) failed to specify the exact method used for random sequence generation, leading to a high risk of bias in this domain. Both RCTs exhibited unclear risks regarding blinding procedures, while the remaining evaluation criteria indicated low risk. The Newcastle-Ottawa Scale was utilized to assess the quality of nonrandomized studies. Among these, three studies achieved a score of 7, whereas the remaining five scored 8 (**Table 2**). Notably, some confounding factors were identified in the comparability between groups and outcome measures across the eight controlled studies, with variations observed in follow-up durations. Despite these issues, the overall quality of the eight non-randomized controlled studies was deemed high.

**Table 3:** Quality assessment according to Cochrane Collaboration Risk of Bias Tool.

	Random sequence generation (selection bias)	Allocation concealment (selection bias)	Blinding of participants and researchers (performance bias)	Blinding of outcome assessment (detection bias)	Incomplete outcome data (attrition bias)	Selective reporting (reporting bias)	Other bias
Abdelaziz et al. (14)	Low risk	Low risk	Unclear risk	Unclear risk	Low risk	Low risk	Low risk
Jacobs et al. (23)	High risk	Low risk	Low risk	Unclear risk	Low risk	Low risk	Low risk

### 3.4 Demographic variables

There were no significant differences between the two groups in terms of body mass index (WMD, -0.063;95% CI, -0.213-0.087; P = 0.410) and neoadjuvant chemotherapy (OR, 0.758;95% CI, 0.531-1.984; P = 0.129); There are some differences between age (WMD, -0.348;95% CI, -0.496, -0.200; P<0.001) and pT ≥3 (OR, 0.732;95% CI, 0.578-0.928; P = 0.01; **Table 4**).

**Table 4:** Demographic variables.

Outcomes	No. studies	No.case	P	WMD or OR (95% CI)	Heterogeneity			
					χ2	df	P	I^2^ (%)
Age	7	301/862	0.000	-0.348(-0.496,-0.200)	4.78	6	0.572	0.0
BMI	6	283/802	0.410	-0.063(-0.213,0.087)	6.75	5	0.240	25.9
pT≥3	7	369/933	0.010	0.732(0.578,0.928)	3.02	6	0.806	0.0
NC	5	238/706	0.129	0.758(0.531,1.984)	1.31	4	0.860	0.0

NC, neoadjuvant chemotherapy; OR, odds ratio; WMD, weighted mean difference.

### 3.5 Functional outcomes

#### 3.5.1 Erectile function

**Figure 2:**
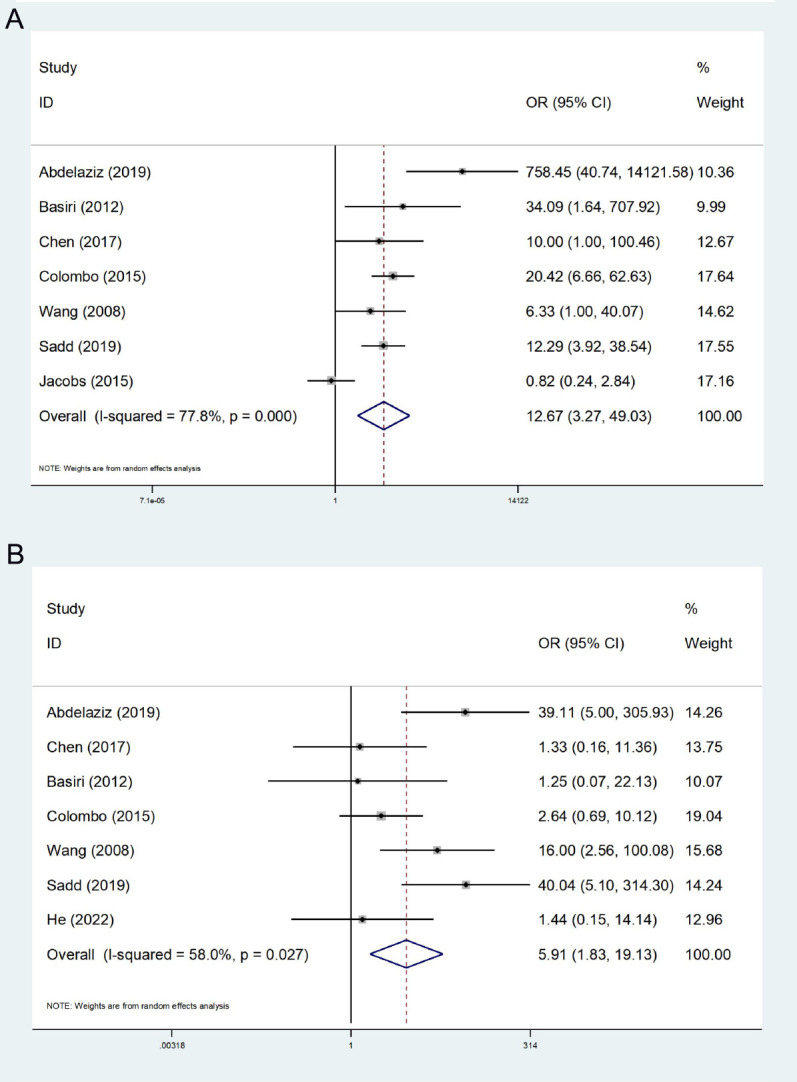
Forest plot and meta-analysis of erectile function **(A)** and daytime continence **(B)**.

A total of 7 studies concerning EF were incorporated (14, 23–28), presenting high heterogeneity among the studies (I^2^ = 77.8%). A random effect model was employed, and the ultimate meta-analysis indicated that EF in the FSRC group was higher than that in the nFSRC group (OR: 12.67; 95% CI, 3.27 - 49.03; P < 0.001, **Figure 2A**).

#### 3.5.2 Daytime continence

In 7 studies regarding daytime urinary continence (14, 24–29), moderate heterogeneity (I^2^ = 58.0%) was noted. Thus, the random effects model was employed for statistical analysis. Our ultimate results indicated that the FSRC group exhibited superior daytime urinary continence compared to the nFSRC group (OR: 5.91; 95% CI, 1.83 - 19.13; P = 0.003, **Figure 2B**).

#### 3.5.3 Nighttime continence

**Figure 3:**
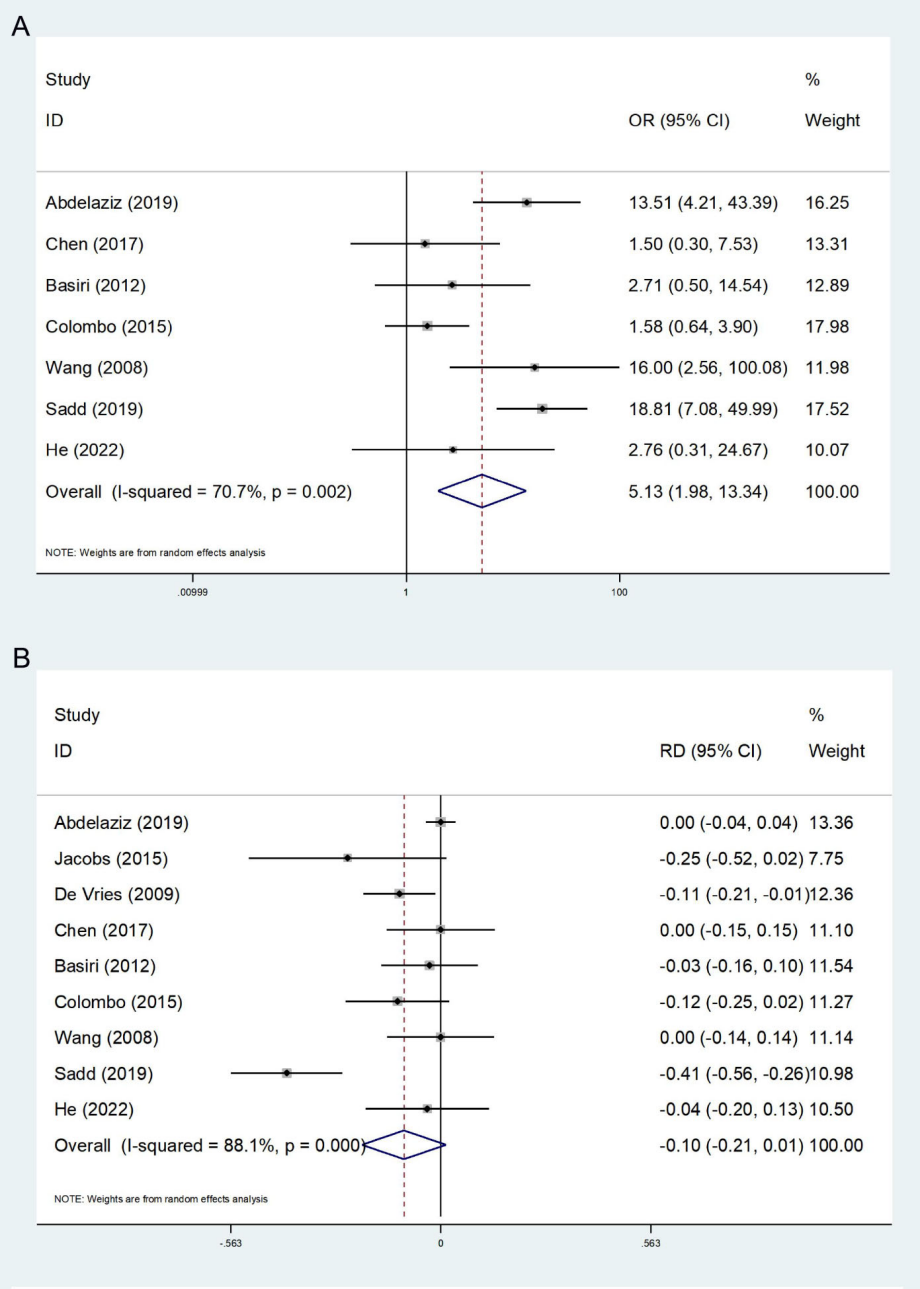
Forest plot and meta-analysis of nighttime continence **(A)** and postoperative incidence of prostate cancer **(B)**.

7 studies report nighttime continence (14, 24–29). The heterogeneity test revealed a high degree of heterogeneity (I^2^ = 70.7%), and a random effects model was used. The final results show that the FSRC group had a lower rate of nighttime continence (OR:5.13; 95% CI, 1.98-13.34; P<0.001, **Figure 3A**).

### 3.6 Oncologic outcomes

#### 3.6.1 Postoperative incidence of prostate cancer

A total of 9 studies reported the incidence of postoperative PCa, encompassing the detection of postoperative pathology and the occurrence of follow-up (13, 14, 23–29). The heterogeneity test indicated a high degree of heterogeneity among the studies (I^2^ = 88.1%); thus, the random effect model was employed in the meta-analysis. The results indicated no statistically significant difference between the FSRC group and the nFSRC group (RD: −0.10; 95% CI, -0.21 - 0.10; P = 0.086, **Figure 3B**). Despite the observed high heterogeneity, which may be attributed to variations in preoperative screening protocols and surgical techniques, the number of included studies suggests that any true effect difference between the two groups is likely to be modest.

#### 3.6.2 Local recurrence

**Figure 4:**
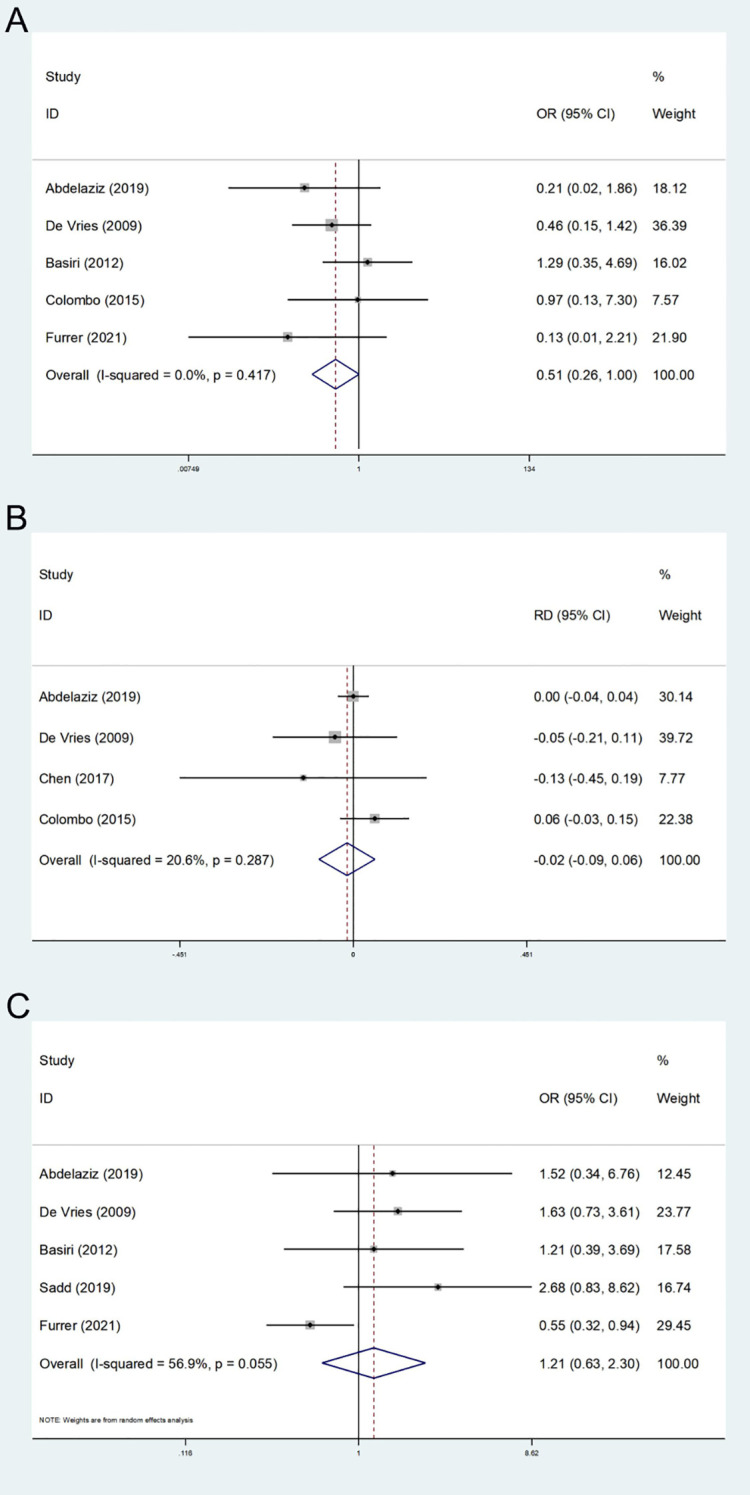
Forest plot and meta-analysis of local recurrence **(A)**, metastatic disease **(B)**, and postoperative survival **(C)**.

Five studies on postoperative local recurrence were encompassed (12–14, 25, 26). The heterogeneity test did not indicate significant heterogeneity (I^2^ = 0.00%). A fixed effect model was employed, and the results revealed that the difference in postoperative local recurrence rates between the two groups was not statistically significant (OR: 0.51; 95% CI, 0.26-1.00; P = 0.052, **Figure 4A**). Although the p-value approached the conventional threshold for statistical significance, suggesting a potential trend toward reduced local recurrence in the FSRC group compared to the nFERC group, the observed effect should be interpreted with caution. Given the limited follow-up duration and small sample sizes in the included studies, the long-term recurrence risk warrants further investigation in well-designed, large-scale trials.

#### 3.6.3 Metastatic disease

A total of 4 studies reported distant metastasis during the follow-up period (13, 14, 24, 26). The heterogeneity test indicated low heterogeneity (I^2^ = 20.6%); therefore, a fixed-effects model was applied. The results showed no statistically significant difference in the risk of postoperative distant metastasis between the two groups (RD: -0.02; 95% CI, -0.09 - 0.06; P = 0.665, **Figure 4B**); However, given the limited follow-up duration and the small number of included studies, these findings do not rule out the possibility of differences in long-term metastatic risk.

#### 3.6.4 Postoperative survival

We assessed the survival status two years after surgery, and a total of five studies were encompassed (12–14, 25, 27). The results of the heterogeneity test (I^2^ = 56.9%) indicated that there was moderate heterogeneity. Utilizing the random effects model, the results indicated no statistically significant difference in two-year survival time between the FSRC group and the nFSRC group (OR: 1.21; 95% CI, 0.63 - 2.30; P = 0.567, **Figure 4C**). Given that the majority of the included studies involved short-term follow-up periods and exhibited heterogeneity, further long-term follow-up studies are warranted to more definitively clarify these findings.

### 3.7 Publication bias and sensitivity analysis

**Figure 5:**
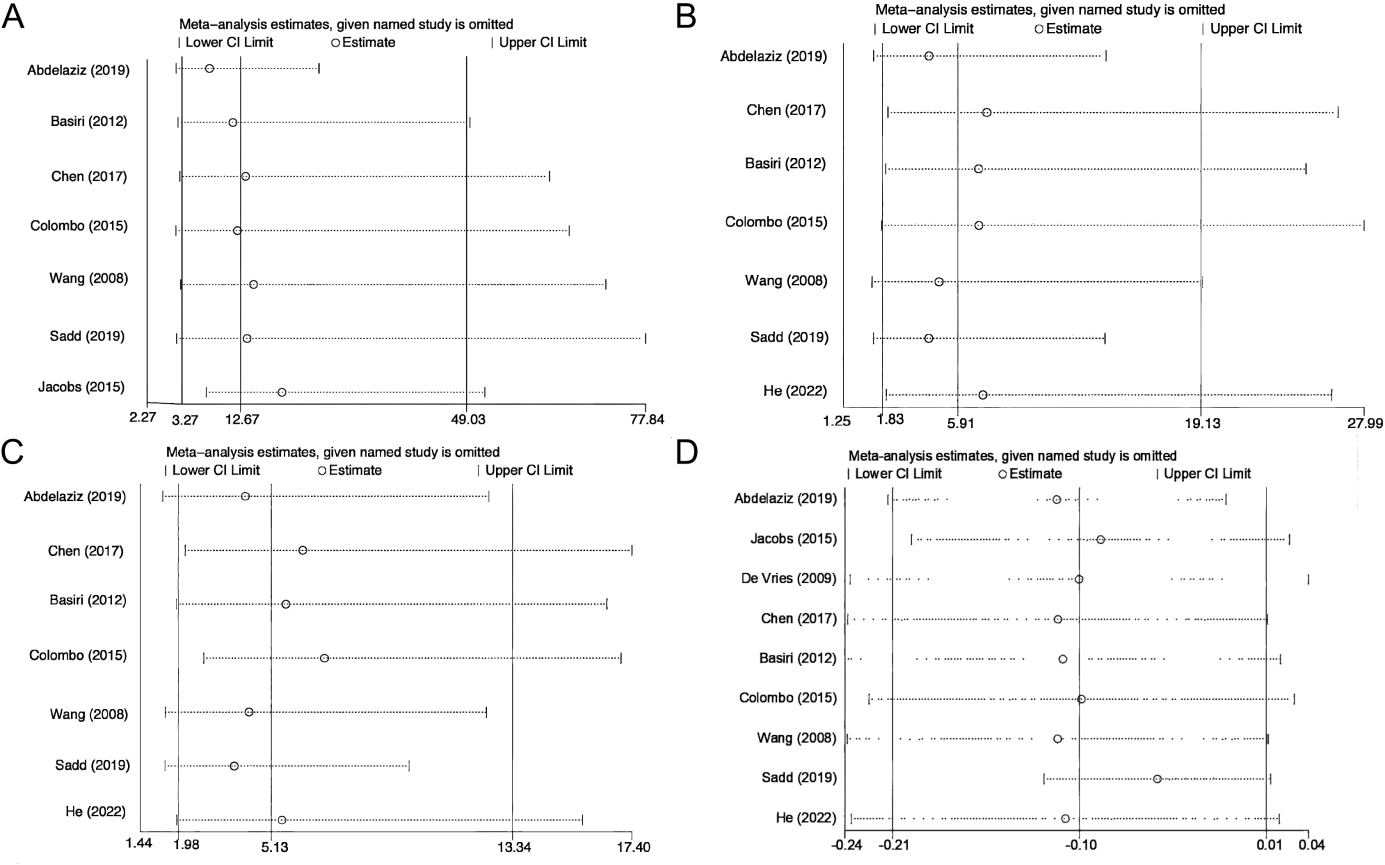
Sensitivity analysis of erectile function **(A)**, daytime continence **(B)**, nighttime continence **(C)**, and postoperative incidence of prostate cancer **(D)**.

Sensitivity analysis was performed on EF, daytime continence, nighttime continence, and postoperative incidence of PCa. We failed to observe relative differences after systematically excluding each study, which attests to the stability of our findings (**Figure 5**). Regarding publication bias, we found no significant publication bias in EF, daytime and nighttime urinary continence, the incidence of postoperative PCa, and postoperative 2-year survival according to Begg’s test (EF: P = 0.764; Daytime urinary continence: P = 1.000; Nighttime urinary continence: P = 1.000; Postoperative PCa: P = 0.707; Local recurrence: P = 0.221; Postoperative 2-year survival: P = 0.462) and Egger’s test (EF: P = 0.99; Daytime urinary continence: P = 0.992; Nighttime urinary continence: P = 0.882; Postoperative PCa: P = 0.425; Local recurrence: P = 0.382; Postoperative 2-year survival: P = 0.113).

### 3.8 Subgroup analyses

Regarding EF, when the sample size was no more than 50, the study type was RCT, and the surgical control was NSC, no significant difference was observed between the FSRC group and the nFSRC group. Concerning daytime and nighttime urinary continence, when the subgroup was European, the study type was retrospective, the sample size was no more than 50, and the surgical control was NSC, there was no significant difference was found between the FSRC group and the nFSRC group. In terms of the incidence of postoperative PCa, except for the subgroup of the Asian region and the difference between the FSRC group and the nFSRC group when the surgical control was NSC, no significant difference was detected in each group. **Table 5** provides detailed statistical data.

**Table 5:** Subgroup analysis.

Outcome	Variable		No. studies	Model	OR/RD (95% CI)	P	I^2^ (%)
Erectile function	Total		7	Random	12.67(3.27,49.03)	<0.001	77.8
	Region	Asia	3	Random	9.99(2.72,36.69)	0.001	0.0
		Europe	2	Random	15.92(7,15,35.44)	<0.001	0.0
		Africa	1	Random	758.45(40.74,14121.58 )	<0.001	–
		North america	1	Random	0.82(0.24,2.84)	0.752	–
	Patients	>50	3	Random	33.50(6.47,173.36)	<0.001	71.9
		≤50	4	Random	4.78(0.91,25.22)	0.065	64.3
	Study type	RCT	2	Random	22.44(0.01,43057.36)	0.420	95.6
		Prospective	2	Random	9.98(2.06,48.26)	0.004	0.0
		Retrospective	3	Random	15.14(7.11,32.25)	<0.001	0.0
	Surgical comparison method	RC	4	Random	29.76(3.56,248.98)	0.002	66.2
		NSC	3	Random	6.00(0.90,40.10)	0.065	87.4
Daytime continence	Total		7	Random	5.91(1.83,19.13)	0.003	58.0
	Region	Asia	4	Random	2.9790.76,11.64)	0.117	33.1
		Europe	2	Random	9.25(0.56,151.69)	0.119	80.8
		Africa	1	Random	39.11(5.00,305.93)	<0.001	–
	Patients	>50	4	Random	8.46(1.48,48.28)	0.016	70.3
		≤50	3	Random	3.57(0.60,21.35)	0.164	47.9
	Study type	RCT	1	Random	39.11(5.00,305.93)	<0.001	–
		Prospective	2	Random	5.73(0.49,67.28)	0.165	53.9
		Retrospective	4	Random	3.78(0.82,17.44)	0.089	60.7
	Surgical comparison method	RC	4	Random	6.55(1.19,36.14)	0.031	59.1
		NSC	2	Random	9.25(0.56,151.69)	0.119	80.8
Nighttime continence	Total		7	Random	5.13(1.98,13.34)	0.001	70.7
	Region	Asia	4	Random	3.52(1.27,9.75)	0.016	21.8
		Europe	2	Random	5.41(0.48,61.41)	0.174	92.5
		Africa	1	Random	13.51(4.21,43.39)	<0.001	–
	Patients	>50	4	Random	6.08(1.54,23.99)	0.010	81.2
		≤50	3	Random	3.80(0.98,14.74)	0.054	47.2
	Study type	RCT	1	Random	13.51(4.21,43.39)	<0.001	–
		Prospective	2	Random	6.33(1.11,36.13)	0.038	49.1
		Retrospective	4	Random	3.52(0.82,15.13)	0.091	80.3
	Surgical comparison method	RC	4	Random	5.66(1.79,17.94)	0.003	54.9
		NSC	2	Random	5.41(0.48,61.41)	0.174	92.5
Postoperative incidence of prostate cancer	Total		9	Random	-0.10(-0.21,0.01)	0.086	88.1
	Region	Asia	4	Random	-0.02(-0.09,0.06)	0.656	0.0
		Europe	3	Random	-0.21(-0.40,-0.02)	0.030	84.7
		Africa	1	Random	0.00(-0.04,0.04)	1.000	–
		North america	1	Random	-0.25(-0.52,0.02)	0.065	–
	Patients	>50	5	Random	-0.13(-0.32,0.06)	0.169	94.1
		≤50	4	Random	-0.04(-0.14,0.06)	0.442	34.7
	Study type	RCT	2	Random	-0.12(-0.63,0.40)	0.656	93.2
		Prospective	3	Random	-0.06(-0.13,0.01)	0.075	3.3
		Retrospective	4	Random	-0.14(-0.34,0.05)	0.157	85.5
	Surgical comparison method	RC	5	Random	-0.03(-0.09,0.04)	0.380	50.6
		NSC	3	Random	-0.26(-0.47,-0.05)	0.014	76.1

## 4 Discussion

### 4.1 Characteristics of FSRC

It is imperative to conduct rigorous preoperative screening and carefully evaluate the indications, particularly focusing on patient age, tumor stage, and grade, and the presence of metastasis. Pyrgidis et al. (46) conducted a retrospective study on the long-term health-related quality of life in BC patients following surgery and found that, among patients over 70 years of age, the combination of RC and ONB reconstruction may adversely affect quality of life. Therefore, careful consideration should be given before performing FSRC in this age group.FSRC modifies traditional surgical techniques by preserving the neurovascular bundles surrounding the prostate as well as the seminal vesicle and vas deferens. Compared with RC, FSRC primarily aims to preserve either the prostate or its capsule, or selectively retain the seminal vesicle and vas deferens during surgery. Specific surgical methods differ in terms of how the prostate is removed and how the neobladder is reconstructed. Currently, preserving the prostatic capsule is more commonly adopted in FSRC in clinical practice. Among the included studies, Abdelaziz et al. (14, 23, 25–29) utilized this technique in seven studies. ONB reconstruction was performed using the terminal ileum or sigmoid colon, followed by anastomosis with the residual capsule. Some studies (43) have explored preoperative transurethral resection of the prostate before proceeding with FSRC; however, the risk of implantation and metastasis remains a topic of debate. Only two studies by De Vries et al. (13, 24) employed PSC, PLND, and bladder resection while preserving the prostate tissue, seminal vesicles, vas deferens, and peripheral neurovascular bundles either entirely or partially. Colombo et al. (12, 26) opted for FSRC that preserved only the seminal vesicle and sexual nerves, while completely excising the prostate and bladder, resulting in significant trauma but adhering strictly to tumor control principles. Salem et al. (44) applied this surgical approach to patients with BC and achieved comparable satisfactory outcomes relative to RC. It is noteworthy that ureteroenteric anastomotic stenosis (UAS) may develop following FSRC. Bizzarri et al. (47) reported that patients with preoperative lower albumin serum levels, lower albumin/fibrinogen ratio, and higher fibrinogen levels are at increased risk of developing UAS, thereby providing a potential basis for patient selection before FSRC.

### 4.2 Functional outcomes

This meta-analysis demonstrates that FSRC may be more efficacious in enhancing postoperative EF and could potentially offer advantages compared to nFSRC. Additionally, we discovered that in contrast to nFSRC, postoperative daytime and nighttime urinary continence was improved. We performed publication bias analysis, sensitivity analysis, and subgroup analysis on the outcomes of the meta-analysis to identify the potential reasons that might influence the results. As of now, no other studies have conducted relevant analyses.

Regarding the preservation of postoperative EF, our study aligns with the outcomes of several previous studies (13, 14, 25–27, 30). The possible mechanism might be associated with the conservation of seminal vesicles and neurovascular bundles during the operation to prevent sexual nerve injury. Nevertheless, the randomized controlled study by Jacobs et al. (23) indicated that there was no significant disparity in the postoperative erectile efficacy between the two groups of PCSC and NSC (50% vs 40%). In the subgroup analysis, we discovered that there was no substantial difference between the FSRC group and the NSC group, which concurred with Jacobs’ results, but the number of studies included in the analysis was limited. Voskuilen et al. (31) demonstrated that 86% of patients in the PSC study maintained EF, which is superior to the results reported after NSC. The preservation rate of EF was 29%-78% after NSC (26, 32), and some patients employed sexual function-improving drugs to enhance EF after the surgery. This also implies that the original nerve was fully preserved; Saad et al. (27) pointed out that without any ED treatment before the surgery, nearly 53% of PCSC patients maintained sexual function without using any drugs, compared with 9% of NSC patients. The latest meta-analysis by Dall et al. (15) also indicated that PCSC is more effective than NSC. Overall, after appropriate selection, FSRC can offer more alternatives for the pursuit of sexual function and a high quality of life after surgery, and holds certain advantages.

Most patients experience urinary incontinence after RC, which might be attributed to the damage to the external urethral sphincter or neurovascular bundle during deep dissection (33). FSRC can better preserve the relevant urinary control structure, and may yield better outcomes (8, 14). Our study suggests that the overall urinary continence of FSRC is superior to nFSRC, and the incidence of daytime urinary incontinence is lower. However, it has been noted that a higher rate of urinary continence is associated with a greater need for catheter insertion secondary to urinary retention (15), which could be caused by strictures at the vesicourethral anastomosis of PCSC and the prostatic urethra. Voskuilen et al. (31) indicated that 95.6% and 70.2% of patients achieved complete recovery of urinary continence during the day and night after PSC, providing better postoperative urinary continence than PCSC and seminal-sparing cysto-prostatectomy (SSC). The randomized controlled trial by Jacobs (23), which aimed at evaluating the control effect, pointed out that there was no difference in urinary continence between PCSC and NSC. Due to the small sample size of the study, the conclusion requires support from more randomized studies with a large sample size. However, Muto et al. (34) indicated that the controllable rate of the PCSC at night was merely 31%, which might be attributed to the disparities in research timing and surgical techniques. Additionally, among RC patients, patients with ONB and nighttime urinary incontinence have lower quality of life and higher depression scores (35). PCSC may enhance the quality of life by improving nighttime sleep and reducing sexual side effects, while Chen et al. (24) indicated that the incidence of nighttime urinary incontinence and the self-catheterization rate in the PCSC group were lower than those in the control group. Furthermore, Volz et al. (48) conducted a propensity score matching analysis over a 4-year follow-up period and found that different types of urinary diversion did not significantly influence disease-specific or general health-related quality of life outcomes, thereby providing indirect support for the functional benefits of FSRC.

### 4.3 Oncologic outcomes

The deterioration of tumors following surgery is a matter of concern for numerous clinicians and patients. Regarding the treatment of malignant tumors, the ultimate objective of any surgical approach is to achieve complete cure or exert maximum control over tumor recurrence and metastasis. Currently, most FSRC procedures, except for the SSC procedure, carry the risk of PCa. In this meta-analysis, we discovered that there was no significant disparity in the incidence of PCa between FSRC and nFSRC. Dall et al. (15) reported in a recent meta-analysis that only 2% of patients who underwent PCSC were identified to have clinically significant postoperative PCa. Nevertheless, these PCa rates are considerably lower than the rate of incidentally detected PCa during RC, which has been reported to be as high as 40% (36). Studies have indicated that the risk of PCa or urothelial cancer continues to decline in appropriately screened patients (23, 27, 37), and there is no clear evidence that the survival time of patients with occult PCa is affected (34). However, some scholars have pointed out that preoperative risk factors are insufficient to accurately predict clinically significant PCa, and the potential oncological risk of PSC must be taken into account (38). Although there is no significant difference in the incidence of postoperative PCa between the two surgical approaches, it is important to note that the long-term risk of prostate cancer following FSRC remains uncertain, underscoring the necessity of preoperative screening. Incidentally detected PCa does not appear to impact patient survival; the surgical method seems to be safe.

As Smith et al. (39) indicated, the proposition of FSRC represents a novel therapeutic approach for BC, which is highly technical and challenging. Nevertheless, the oncological control outcomes remain controversial and warrant further investigation. The findings of our meta-analysis demonstrated that there was no statistically significant difference in tumor recurrence and metastasis between postoperative FSRC and nFSRC, aligning with the results of certain recent studies (14, 15, 27, 37). Voskuilen et al. (31) noted that the local BC recurrence rate of PSC was 11%. Simone et al. (40) also remarked that patients with T2G3 tumors exhibited extremely poor oncological outcomes, with eight out of ten patients experiencing recurrence; this might be associated with the belated preoperative staging and grading. Botto et al. (41) et al. discovered an augmented risk of distant metastasis (17.6%) in patients who underwent PCSC without local recurrence, suggesting the existence of micrometastatic disease before the intervention. In another study, among 100 patients who underwent PCSC, five had local recurrence and 31 had distant metastasis, but among these patients, 17 had T3 disease and/or nodal disease (42), indicating that the stage rather than the surgery was determining the outcome. Given the limited follow-up duration and the small number of included studies, the current findings do not preclude the potential for differences in long-term recurrence and metastasis; more data are required in the future. Based on the current results, the tumor control of the FSRC is not inferior to that of nFSRC.

The postoperative survival duration of patients is also one of the metrics for evaluating surgical outcomes. We compared the 2-year survival period of FSRC with nFSRC. This is in line with the findings of numerous previous studies (14, 23–28), and the FSRC did not harm the postoperative survival of patients, which is approximately in accordance with nFSRC.

### 4.4 Subgroup analysis

We observed a significant degree of heterogeneity in the outcomes of EF (I^2^ = 77.8%). Subgroup analysis suggests that this heterogeneity may be attributed to small sample sizes, varying study designs, and differing surgical controls. Additionally, differences in follow-up periods could contribute to variations in postoperative EF. High heterogeneity was also noted in the analysis of nocturnal urinary continence (I^2^ = 70.7%). Subgroup analysis revealed that heterogeneity in research findings might be influenced by variations in surgical techniques, geographical regions of the included populations, and the retrospective nature of some studies. Furthermore, differences in postoperative evaluation methods likely contributed to this heterogeneity. For the incidence of postoperative PCa, we identified high heterogeneity as well. Subgroup analysis indicated that disparities in study regions, methodologies, sample sizes, and control groups could account for the heterogeneity in results. In conclusion, these heterogeneous factors reflect variations in medical practices, demographic characteristics, and socioeconomic backgrounds. These include the degree of standardization in surgeons’ experience and techniques, differences in pelvic floor anatomy among the study population, preoperative comorbidities, and postoperative rehabilitation protocols.

Based on our review of the included studies, it is evident that FSRC clinical research, whether RCTs or non-randomized controlled trials (nRCTs), requires adherence to rigorous principles in clinical research design. Careful planning and detailed protocols should be established before study initiation, with active management of confounding factors, rational allocation strategies, and assurance of complete follow-up data. The implementation process must also strictly adhere to the planned protocol to ensure accurate and reliable clinical research outcomes. Despite the heterogeneity observed in some studies, our overall findings provide valuable insights.

### 4.5 Strengths and limitations

#### 4.5.1 Surgical methods

FSRC is a modified operation of RC. After RC, patients with ED, urinary continence dysfunction, and other complications, especially young patients, will permanently lose their reproductive function and seriously reduce the quality of life of patients with BC. FSRC preserves the relevant male reproductive structure, including the prostate or seminal vesicle or prostate capsule, and improves EF and urinary continence function after surgery, which has been supported by many controlled studies and clinical observations (6, 23–28, 34, 39). However, some researchers have noted that while postoperative complications have decreased, the risk of recurrence of urethral and pelvic tumors, as well as PCa, may have increased (14, 40, 45), and the treatment choice is still controversial. A sufficient volume of a particular surgical procedure is generally associated with improved outcomes in terms of mortality and may also contribute to better functional recovery. Pyrgidis et al. (49) conducted a retrospective analysis of data from all hospitalized patients who underwent BC surgery in Germany, demonstrating that centralization of RC not only reduces hospitalization-related morbidity and mortality but also shortens length of stay and lowers healthcare costs. A threshold of at least 50 RC procedures per year per institution was identified as necessary to achieve optimal outcomes. These findings suggest that variations in treatment outcomes may be influenced by both the treating hospital and its procedural volume. Meanwhile, Milling et al. (50) demonstrated through a cross-sectional study that RC surgery significantly reduces female patients’ satisfaction with sexual function, suggesting that women experience similar postoperative challenges. Our findings may provide a preliminary foundation for future research on female FSRC.

Overall, this study provides evidence in support of the efficacy and safety of FSRC in the treatment of BC.Although FSRC appears promising, there remains insufficient evidence to support its routine clinical implementation, except in carefully selected young patients.

#### 4.5.2 Research result

The main advantage of this meta-analysis lies in the fact that we conducted sensitivity analysis and subgroup analysis for each outcome index. Despite the limited number of included studies and the relatively small sample size of each study, the stability and heterogeneity of the corresponding outcome were explored to a certain extent. In the subgroup analysis, it was discovered that there were some disparities in the outcome indicators when classified based on sample size, study type, control group, and study area, which could also offer some assistance for the design of related research in the future.

We accomplished this meta-analysis in accordance with the strict guidelines of PRISMA; however, certain limitations persist. Firstly, heterogeneity was evident in some outcome measures, such as EF, daytime and nighttime urinary continence, the incidence of postoperative PCa, and 2-year survival after surgery. We identified that the heterogeneity could be attributed to the selection of surgical modalities, inconsistent preoperative baseline characteristics, variations in the definitions of each index, and surgeon proficiency, among other factors. Therefore, our conclusions should be interpreted cautiously. Secondly, some studies were small-scale, retrospective, and non-randomized, entailing a certain risk of bias and confusion, which might influence the overall quality of evidence. Thirdly, several factors could affect the results, including differences in surgical methods, disparities in control groups, and tumor staging.

## 5 Conclusion

In this meta-analysis, FSRC with ONB is capable of enhancing postoperative EF and urinary continence. There was no statistically significant disparity in the improvement of postoperative survival time between the two groups. It remains unclear whether there are differences in tumor recurrence, metastasis, and PCa occurrence between the two groups. Overall, FSRC may offer a functional benefit in certain patient populations without substantially compromising oncologic safety, thereby supporting its continued evaluation in clinical practice. However, additional high-quality randomized controlled trials are required before broad implementation can be recommended. Although the existing evidence remains limited, current findings provide a compelling rationale for further investigation of this approach in highly selected patient cohorts.

## Data Availability

The original contributions presented in the study are included in the article/supplementary material. Further inquiries can be directed to the corresponding author.
